# Associations between estimated glomerular filtration rate and cardiac biomarkers

**DOI:** 10.1002/jcla.23336

**Published:** 2020-04-16

**Authors:** Lu Pang, Zhe Wang, Zi‐Long Zhao, Qi Guo, Chen‐Wei Huang, Jia‐Lin Du, Hong‐Yun Yang, Hai‐Xia Li

**Affiliations:** ^1^ Department of Clinical Laboratory Peking University First Hospital Beijing China; ^2^ Department of Infection Control China Academy of Chinese Medical Sciences Xiyuan Hospital Beijing China; ^3^ Department of Clinical Laboratory Capital Institute of Pediatrics Beijing China

**Keywords:** cardiology, renal disease, troponin

## Abstract

**Background:**

Chronic kidney disease (CKD) is associated with an increased cardiovascular disease (CVD) mortality risk. Elevation of cardiac biomarkers in patients with renal dysfunction is ambiguous in the diagnosis of CVD. The purpose of this study was to investigate the associations between estimated glomerular filtration rate (eGFR) and cardiac biomarkers, and the influence of renal dysfunction on the cardiac biomarkers.

**Methods:**

We examined the cross‐sectional associations of eGFR with cardiac troponin I (cTnI), creatine kinase (CK), CK‐MB, lactic dehydrogenase (LDH), hydroxybutyrate dehydrogenase (HBDH), and brain natriuretic peptide (BNP) in 812 adults and 215 child. Spearman correlation and logistic regression analysis were performed to evaluate the associations.

**Results:**

For adults, lower eGFR _CKD‐EPI_ had significantly higher cTnI, CK‐MB, LDH, HBDH, and BNP. There were negative correlations between eGFR_CKD‐EPI_ and cTnI, CK‐MB, LDH, HBDH, and BNP. After adjustment for potential confounders, as compared with eGFR_CKD‐EPI_ ≥ 90 mL/min/1.73 m^2^, eGFR_CKD‐EPI_ < 60 mL/min/1.73 m^2^ remained associated with a 2.83 (1.08‐7.41) [ratio (95% CI)] times higher cTnI and a 6.50 (2.32‐18.22) [ratio (95% CI)] times higher HBDH. For child, lower eGFR_Schwartz_ had significant higher CK and CK‐MB. There were negative correlations between eGFR_Schwartz_ and CK, and eGFR_Schwartz_ and CK‐MB. After adjustment for potential confounders, as compared with eGFR_Schwartz_ ≥ 90 mL/min/1.73 m^2^, eGFR_Schwartz_ < 90 mL/min/1.73 m^2^ revealed no significant higher CVD biomarkers.

**Conclusion:**

Reduced eGFR is associated with elevated cTnI and HBDH among adults without clinically evident CVD, but not child.

## 1 INTRODUCTION

For the patients with chronic kidney disease (CKD), which is defined as an estimated glomerular filtration rate (eGFR) < 60 mL/min/1.73 m^2^ for 3 months, the most frequently encountered cause of death is cardiovascular diseases (CVD),^1^, ^2^, ^3^ such as myocardial infarction and heart failure. A large cohort study comprising >130 000 elderly participates showed that increased incidence of cardiovascular events was related to the renal insufficiency.^4^

The diagnosis of CVD is usually based on clinical manifestation, electrocardiographic (ECG) changes, and positive cardiac biomarkers. Cardiac biomarkers, such as cardiac troponin I (cTnI),^5^, ^6^ creatine kinase (CK),^6^ CK‐MB,^7^ lactic dehydrogenase (LDH),^8^ hydroxybutyrate dehydrogenase (HBDH),^8^ and brain natriuretic peptide (BNP),^9^ play important roles in the diagnosis of CVD. Among them, cTnI is a sensitive and specific marker of myocardium damage and is a widely used predictor of cardiovascular events^10^ and BNP has widespread utility as an adjunct to CVD diagnosis and management.^9^

It is well known that cardiac biomarkers are often increased in patients with impaired renal function, which made the interpretation of cTnI, CK, CK‐MB, LDH, HBDH, and BNP is ambiguous and the diagnosis of CVD is challenging in patients with impaired renal function.^11^ Information on the association between cardiac biomarkers and eGFR is currently limited, and the elevation of the cardiac biomarkers at a given eGFR is not well clarified, especially in child. For example, Remy et al^12^ found that eGFR 60 to <90 mL/min/1.73 m^2^ was associated with a 1.19 (1.12‐1.27) [ratio (95%CI)] times higher cTnI, but Tuncay et al^13^ found that there was no significant relationship between eGFR and cTnI. Thus, the associations between eGFR and cardiac biomarkers need to be further evaluated.

In view of the above, we examined whether eGFR was associated with cTnI, CK, CK‐MB, LDH, HBDH, and BNP in Chinese population, including both adults and child.

## 2 MATERIALS AND METHODS

### 2.1 Study population

**FIGURE 1:**
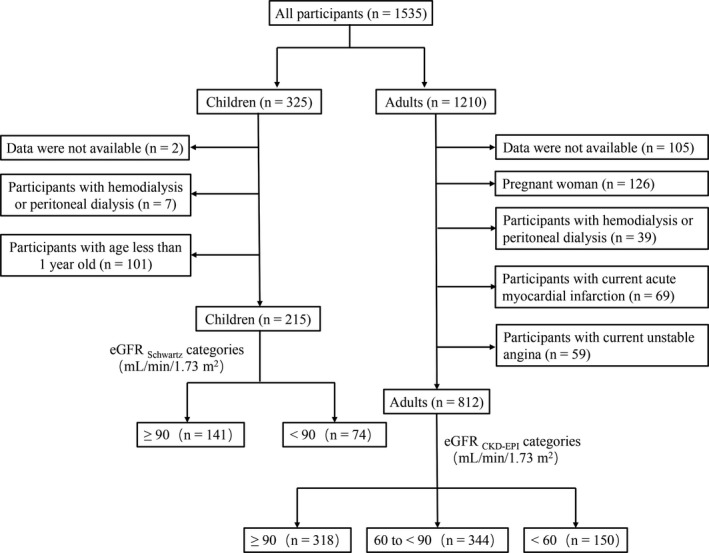
Schematic illustration of patient recruitment. Abbreviation: CKD‐EPI, the Chronic Kidney Disease Epidemiology Collaboration; eGFR, estimated glomerular filtration rate; MDRD, the Modification of Diet in Renal Disease

In total, 1210 adults and 325 child who performed serum creatinine measurement and cardiac biomarkers measurement simultaneously between December 2018 and February 2019 in Peking University First Hospital were initially reviewed retrospectively. Based on the medical record review, 398 adults were excluded due to unavailable data, pregnancy, hemodialysis, peritoneal dialysis, current acute myocardial infarction, and current unstable angina, and 110 child were excluded due to unavailable data, hemodialysis, peritoneal dialysis, and <1 year old. Finally, 812 adults and 215 child were included. The detailed flowchart of patient recruitment was shown in Figure 1. The research was in compliance with the Declaration of Helsinki and approved by the ethics committee of Peking University First Hospital (reference number: 1381).

### 2.2 Biochemistry biomarkers

Serum creatinine (Jaffe method) was measured by AU5800 automatic biochemical analyzer (Beckman Coulter, Inc). For adults, eGFR was calculated with the Chronic Kidney Disease Epidemiology Collaboration (CKD‐EPI)equation recommended by the Kidney Disease Improving Global Outcomes (eGFR_CKD‐EPI_)^14^ and the modified Modification of Diet in Renal Disease (MDRD) equation (eGFR _MDRD_).^15^ For child, eGFR was calculated with the Schwartz formula (eGFR_Schwartz_).^16^

Coupled multienzyme method (CK), lactic acid method (LDH), and α‐ketobutyric acid method (HBDH) were measured by AU5800 automatic biochemical analyzer (Beckman Coulter, Inc). CK‐MB, cTnI, and BNP were measured by chemiluminescent enzyme immunoassay with UniCel Dxl 800 Access automatic biochemical analyzer (Beckman Coulter, Inc).

### 2.3 Covariates

We collected data on age, gender, body mass index (BMI), smoking behavior, alcohol behavior, ST‐T wave abnormalities of resting 12‐lead ECG, previous coronary heart disease (CHD), previous CHD surgeries, other heart diseases, hypertension, diabetes, antihypertensive medications, lipid‐modifying medications, antiplatelet drugs, triglyceride (TG), low‐density lipoprotein‐cholesterol (LDL‐C), high‐density lipoprotein‐cholesterol (HDL‐C), and urea. For adults, smoking and alcohol behavior were categorized into never, former, and current. All the former had ceased smoking or drinking for at least 12 months. For child, smoking and alcohol behavior were categorized into never and current. Previous CHD surgeries include percutaneous coronary intervention and coronary artery bypass grafting. Other heart diseases include cardiac arrhythmia, congenital cardiovascular diseases, cardiomyopathy, rheumatic heart disease, valvular heart disease, infective endocarditis, and myocarditis. Antihypertensive medications include angiotensin‐converting enzyme inhibitors, angiotensin receptor blockers, calcium ion antagonists, and β‐adrenoceptor blockers. Lipid‐modifying medications include statins, probucol, and acipimox. Antiplatelet drugs include aspirin, clopidogrel, and ticagrelor. TG (enzymatic method), LDL‐C (surfactant method), HDL‐C (surfactant method), and urea (urease method) were measured by AU5800 automatic biochemical analyzer (Beckman Coulter, Inc). LDL‐C/HDL‐C was calculated by dividing LDL‐C by HDL‐C.

### 2.4 Statistical analyses

Data were statistically analyzed by the SPSS software version 21.0 for Windows (IBM). Graphs were prepared using GraphPad Prism version 6.0 (GraphPad Software). We have tried logarithmic transformation for data with non‐Gaussian distribution. Finally, data are represented as means ± standard deviations for Gaussian distribution and medians (interquartile ranges) for non‐Gaussian distribution and n (%) for categorical data. Student's *t* test and one‐way ANOVA test were used to compare differences between continuous data with Gaussian distribution. Mann‐Whitney *U* test and Kruskal‐Wallis test were used to compare differences between continuous data with non‐Gaussian distribution. Chi‐square (*χ* ^2^) test was used to compare differences between categorical data. Spearman correlation was performed between cardiac biomarkers and age or eGFR. A two‐tailed *P* value < .05 was considered statistically significant.

Associations of eGFR_CKD‐EPI_ and eGFR_Schwartz_ with cTnI, CK, CK‐MB, LDH, HBDH, and BNP were evaluated with logistic regression analysis. eGFR _CKD‐EPI_ and eGFR_Schwartz_ were analyzed as categorical variable (for eGFR_CKD‐EPI_: ≥90, 60 to <90 and <60 mL/min/1.73 m^2^; for eGFR_Schwartz_: ≥90 and <90 mL/min/1.73 m^2^). According to the manufactory and laboratory verification, the elevated cutoff points of cTnI, CK, CK‐MB, LDH, HBDH, and BNP were determined as 0.03 ng/mL, 195 IU/L, 5 ng/mL, 240 IU/L, 220 IU/L, and 100 pg/mL, respectively. We have deleted the missing value because the number of missing values is small. We adjusted for potential covariates as follows: Model 1: unadjusted model; Model 2: age, gender, BMI, smoking, and alcohol behavior; Model 3: model 2 + urea, TG, LDL‐C/HDL‐C, ST‐T wave abnormalities of ECG, previous CHD, previous CHD surgeries, hypertension, and diabetes; Model 4: model 3 + antihypertensive medications, lipid‐modifying medications, antiplatelet drugs, and other heart diseases. Furthermore, we replaced eGFR_CKD‐EPI_ by eGFR_MDRD_ to repeat the analysis in adult participants.

## 3 RESULTS

### 3.1 Characteristics of the study population

Altogether, 812 adults and 215 child were included in this study finally. For adults, the mean age of participants was 60.6 ± 16.5 years, and 58.3% were male. For child, the mean age of participants was 7.9 ± 5.0 years, and 52.1% were boys.

The clinical characteristics of the adults and child population stratified according to eGFR_CKD‐EPI_ and eGFR_Schwartz_ categories were shown in Table 1 and Table S1, respectively. The median eGFR_CKD‐EPI_ was 80.24 mL/min/1.73 m^2^. Most participants had an eGFR_CKD‐EPI_ ≥ 90 mL/min/1.73 m^2^ (39.1%) or 60 to <90 mL/min/1.73 m^2^ (42.4%), while 18.5% had eGFR_CKD‐EPI_ < 60 mL/min/1.73 m^2^. The median eGFR_Schwartz_ was 99.93 mL/min/1.73 m^2^. 65.6% participants had an eGFR_Schwartz_ ≥ 90 mL/min/1.73 m^2^, and 34.4% had an eGFR_Schwartz_ < 90 mL/min/1.73 m^2^. In adults, participants with lower eGFR had a worse CVD risk profile, such as hypertension and diabetes. Progressively higher eGFR _CKD‐EPI_ categories were significantly associated with higher rates of previous CHD, previous CHD surgeries, medications use, and ST‐T wave abnormalities of ECG and higher urea.

**TABLE 1:** Clinical characteristics of the adults participants stratified according to eGFR_CKD‐EPI_ categories^a^

	Study population	eGFR_CKE‐EPI_ categories (mL/min/1.73 m^2^)			*P* values^b^
		≥90	60 to <90	<60	
Number	812	318 (39.1%)	344 (42.4%)	150 (18.5%)	
Demographics					
Age (y)	60.6 ± 16.5	50.4 ± 13.6	67.3 ± 12.9	66.5 ± 18.3	<.001
Gender					
Male	473 (58.3%)	196 (61.6%)	190 (55.2%)	87 (58.0%)	.248
Female	339 (41.7%)	122 (38.4%)	154 (44.8%)	63 (42.0%)	
BMI (kg/m^2^)	24.66 ± 3.76	24.66 ± 3.92	24.69 ± 3.48	24.58 ± 4.04	.960
Lifestyle variables					
Smoking behavior					
Never	532 (65.5%)	197 (61.9%)	237 (68.9%)	98 (65.3%)	.006
Former	118 (14.5%)	38 (11.9%)	52 (15.1%)	28 (18.7%)	
Current	162 (20.0%)	83 (26.2%)	55 (16.0%)	24 (16.0%)	
Alcohol behavior					
Never	598 (73.7%)	209 (65.7%)	274 (79.7%)	115 (76.7%)	<.001
Former	49 (6.0%)	19 (6.0%)	20 (5.8%)	10 (6.7%)	
Current	165 (20.3%)	90 (28.3%)	50 (14.5%)	25 (16.6%)	
Lipid					
TG (mmol/L)	1.34 (0.88‐1.99)	1.35 (0.84‐2.10)	1.29 (0.90‐1.83)	1.48 (1.02‐2.34)	.034
LDL‐C/HDL‐C	2.40 (1.84‐3.15)	2.45 (1.97‐3.21)	2.25 (1.76‐2.96)	2.49 (1.83‐3.49)	.027
Medical history					
Previous CHD	152 (18.7%)	28 (8.8%)	84 (24.4%)	40 (26.7%)	<.001
Previous CHD surgeries ^c^	77 (9.5%)	14 (4.4%)	41 (11.9%)	22 (14.7%)	<.001
Other heart diseases ^d^	157 (19.3%)	40 (12.6)	82 (23.8%)	35 (23.3%)	<.001
Hypertension	399 (49.1%)	114 (35.8%)	183 (53.2%)	102 (68.0%)	<.001
Diabetes	222 (27.3%)	76 (23.9%)	87 (25.3%)	59 (39.3%)	.001
Medications					
Antihypertensive medications^e^	376 (46.3%)	107 (33.6%)	173 (50.3%)	96 (64.0%)	<.001
Lipid‐modifying medications^f^	194 (23.9%)	62 (19.5%)	87 (25.3%)	45 (30.0%)	.033
Antiplatelet drugs^g^	136 (16.7%)	35 (11.0%)	67 (19.5%)	34 (22.7%)	.001
ST‐T wave abnormalities of ECG	79 (9.7%)	25 (7.9%)	36 (10.5%)	18 (12.0%)	.308
Kidney biomarkers					
Creatine (µmol/L)	95.29 ± 87.80	66.24 ± 12.33	80.62 ± 13.78	190.53 ± 172.53	<.001
eGFR_CKE‐EPI_ (mL/min/1.73 m^2^)	80.24 ± 25.26	102.90 ± 11.19	77.04 ± 8.43	39.52 ± 15.95	<.001
eGFR_MDRD_ (mL/min/1.73 m^2^) ^h^	89.25 ± 37.65	114.88 ± 34.21	77.42 ± 8.24	38.67 ± 16.50	<.001
Urea (mmol/L)	5.44 (4.35‐6.99)	4.75 (3.79‐5.67)	5.39 (4.43‐6.61)	10.18 (7.65‐17.40)	<.001
Cardiac biomarkers					
cTnI (ng/mL)	0.004 (0.001‐0.009)	0.002 (0.001‐0.005)	0.004 (0.001‐0.009)	0.010 (0.005‐0.034)	<.001
CK (IU/L)^i^	68 (45‐104)	66 (43‐102)	67 (47‐99)	76 (45‐124)	.207
CK‐MB (ng/mL)^j^	1.1 (0.7‐1.8)	0.9 (0.6‐1.4)	1.2 (0.8‐1.9)	1.5 (1.0‐2.3)	<.001
LDH (IU/L)^k^	172 (147‐208)	163 (141‐197)	176 (151‐207)	185 (152‐256)	<.001
HBDH (IU/L)^l^	132 (113‐161)	125 (109‐152)	136 (119‐160)	144 (118‐209)	<.001
BNP (pg/mL)^m^	47 (21‐133)	27 (14‐60)	62 (28‐147)	138 (54‐381)	<.001

Abbreviations: BMI, body mass index; BNP, brain natriuretic peptide; CHD, coronary heart disease; CK, creatine kinase; cTnI, cardiac troponin I; ECG, electrocardiogram; eGFR, estimated glomerular filtration rate; HBDH, hydroxybutyrate dehydrogenase; HDL‐C, high‐density lipoprotein‐cholesterol; LDH, lactic dehydrogenase; LDL‐C, low‐density lipoprotein‐cholesterol; MDRD, The Modification of Diet in Renal Disease; TG, triglyceride.

^a^ Data are represented as means ± standard deviations for Gaussian distribution, medians (interquartile ranges) for non‐Gaussian distribution, and n (%) for categorical data.

^b^ *P* values for the comparison of participants across the eGFR categories were calculated with the one‐way ANOVA test for Gaussian distributed data, Kruskal‐Wallis test for non‐Gaussian distribution, and chi‐square (χ^2^) test for categorical data.

^c^ CHD surgeries include percutaneous coronary intervention and coronary artery bypass grafting.

^d^ Other heart diseases include cardiac arrhythmia, congenital cardiovascular diseases, cardiomyopathy, rheumatic heart disease, valvular heart disease, infective endocarditis, and myocarditis.

^e^ Antihypertensive medications include angiotensin‐converting enzyme inhibitors, angiotensin receptor blockers, calcium ion antagonists, and β‐adrenoceptor blockers.

^f^ Lipid‐modifying medications include statins, probucol, and acipimox.

^g^ Antiplatelet drugs include aspirin, clopidogrel, and ticagrelor.

^h^ For eGFR calculated by MDRE equation, there are 393, 287, and 132 participants in three eGFR categories (≥90, 60 to <90, <60 mL/min/1.73 m^2^).

^i^ Data available for 754 participants, including 299, 320, and 135 participants in three eGFR categories (≥90, 60 to <90, <60 mL/min/1.73 m^2^).

^j^ Data available for 763 participants, including305, 321, and 137 participants in three eGFR categories (≥90, 60 to <90, <60 mL/min/1.73 m^2^).

^k^ Data available for 765 participants, including 306, 321, and 138 participants in three eGFR categories (≥90, 60 to <90, <60 mL/min/1.73 m^2^).

^l^ Data available for 740 participants, including 295, 316, and 129 participants in three eGFR categories (≥90, 60 to <90, <60 mL/min/1.73 m^2^).

^m^ Data available for 719 participants, including 281, 299, and 139 participants in three eGFR categories (≥90, 60 to <90, <60 mL/min/1.73 m^2^).

Figure S1 showed the distribution of CVD biochemistry biomarkers in all the participants. According to the cutoff points, 9.0%, 7.3%, 2.7%, 23.8%, 14.1%, and 28.6% patients had elevated cTnI, CK, CK‐MB, LDH, HBDH, and BNP, respectively. Spearman correlation analysis showed positive correlations between age and cTnI (*r* = .391; *P* < .001), CK‐MB (*r* = .310; *P* < .001), LDH (*r* = .120; *P* = .001), HBDH (*r* = .130; *P* < .001), and BNP (*r* = .472; *P* < .001) in adult participant (Figure S2). For child, there were negative correlations between age and CK (*r* = −0.119; *P* = .008), CK‐MB (*r* = −.490, *P* < .001), LDH (*r* = −.542, *P* < .001), and HBDH (*r* = −.603; *P* < .001; Figure S3).

### 3.2 Association between eGFR and CVD biochemistry biomarkers

**FIGURE 2:**
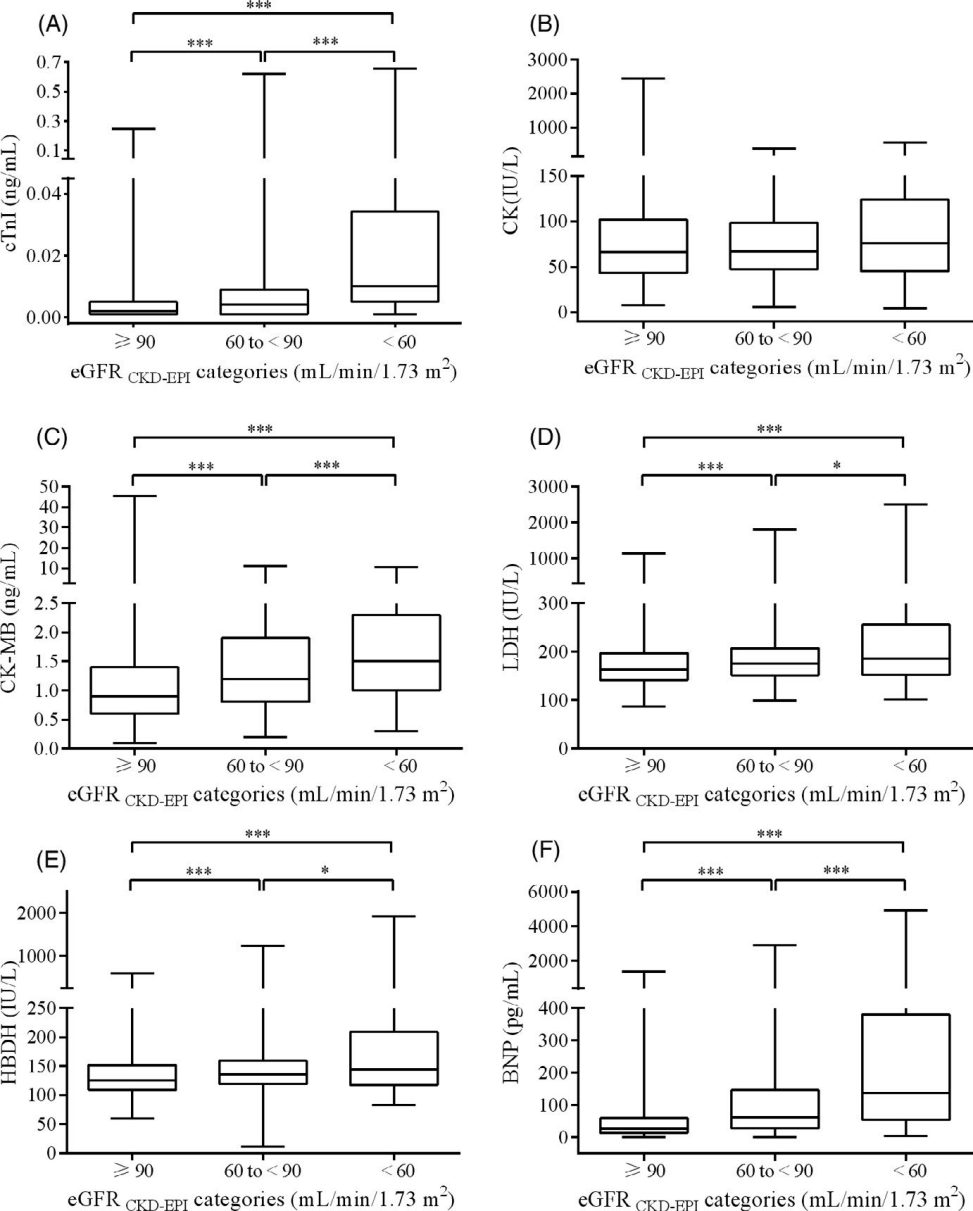
The boxplot of cTnI (A), CK (B), CK‐MB (C), LDH (D), HBDH (E), and BNP (F) stratified according to eGFR_CKD‐EPI_ categories in the adult participants. *P* values for the comparison of participants between the eGFR_CKD‐EPI_ categories were calculated with the Mann‐Whitney *U* test. *P < .05; **P < .01; ***P < .001; Abbreviation: BNP, brain natriuretic peptide; CK, creatine kinase; cTnI, cardiac troponin I; eGFR, estimated glomerular filtration rate; HBDH, hydroxybutyrate dehydrogenase; LDH, lactic dehydrogenase

**FIGURE 3:**
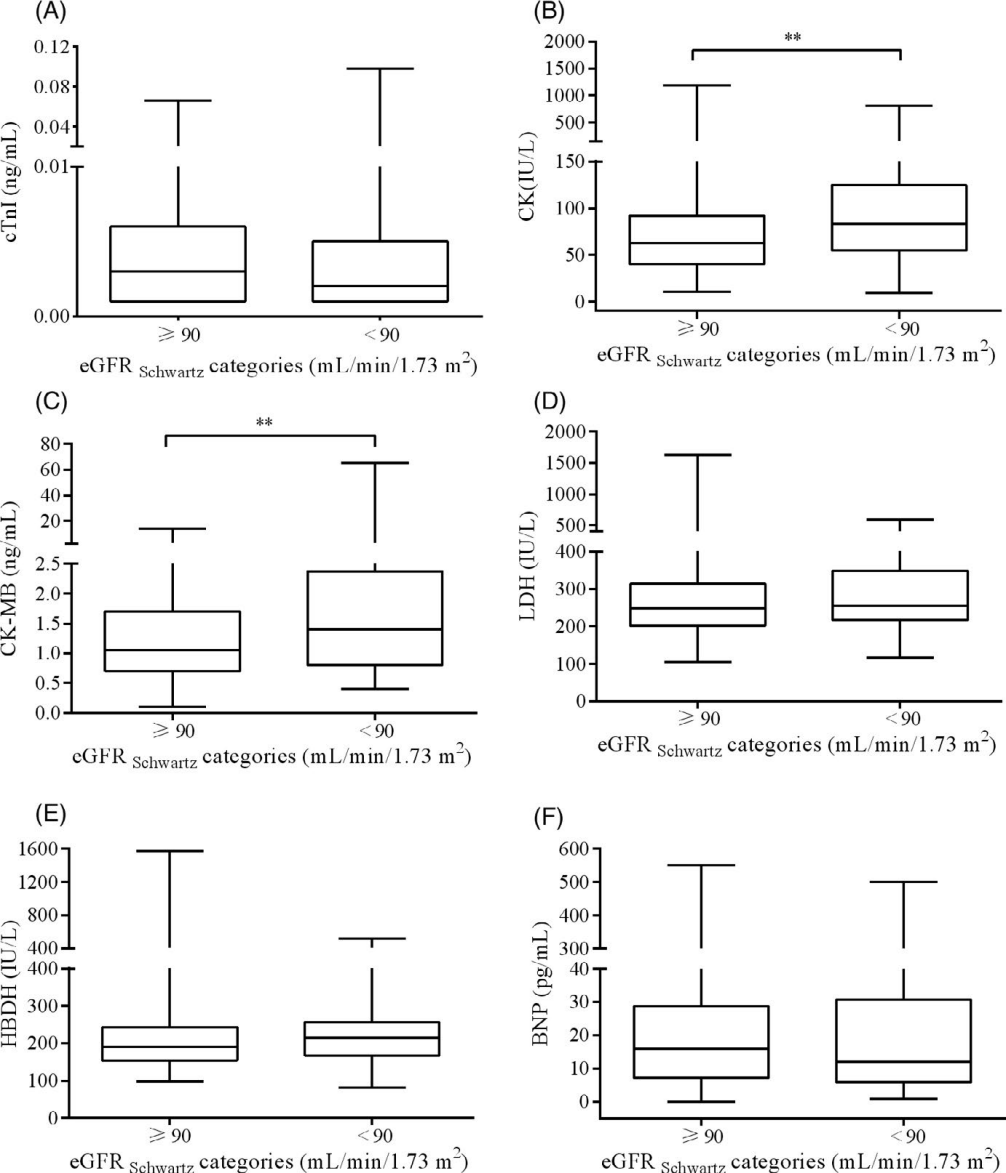
The boxplot of cTnI (A), CK (B), CK‐MB (C), LDH (D), HBDH (E), and BNP (F) stratified according to eGFR_Schwartz_ categories in the child participants. P values for the comparison of participants between the eGFR_Schwartz_ categories were calculated with the Mann‐Whitney *U* test. *P < .05; **P < .01; ***P < .001; Abbreviation: BNP, brain natriuretic peptide; CK, creatine kinase; cTnI, cardiac troponin I; eGFR, estimated glomerular filtration rate; HBDH, hydroxybutyrate dehydrogenase; LDH, lactic dehydrogenase

Participants with lower eGFR _CKD‐EPI_ had significantly higher cTnI, CK‐MB, LDH, HBDH, and BNP (Table 1 and Figure 2). For child, lower eGFR_Schwartz_ had significantly higher CK and CK‐MB (Table S1 and Figure 3).

Spearman correlation analysis showed negative correlations between eGFR _CKD‐EPI_ and cTnI (*r* = −.420; *P* < .001), CK‐MB (*r* = −.276; *P* < .001), LDH (*r* = −.193; *P* < .001), HBDH (*r* = −0.215; *P* < .001), and BNP (*r* = −0.450; *P* < .001) (Table 2 and Figure S4). For child, there were negative correlations between eGFR_Schwartz_ and CK (*r* = −.263; *P* < .001) and eGFR_Schwartz_ and CK‐MB (*r* = −.190; *P* = .006) (Table 3 and Figure S5).

**TABLE 2:** Relationships between cardiac biomarkers and eGFR_CKD‐EPI_ categories

Cardiac biomarkers	Total population		eGFR_CKE‐EPI_ categories (mL/min/1.73 m^2^)					
			≥90		60 to <90		<60	
	*r*	*P* value	*r*	*P* value	*r*	*P* value	*r*	*P* value
cTnI (ng/mL)	−.420	<.001	−.073	.195	−.159	.003	−.147	.074
CK (IU/L)	−.072	.047	−.086	.140	−.031	.579	.095	.027
CK‐MB (ng/mL)	−.276	<.001	−.201	<.001	−.103	.064	.006	.947
LDH (IU/L)	−.193	<.001	−.143	.013	−.018	.743	−.212	.012
HBDH (IU/L)	−.215	<.001	−.146	.012	−.052	.354	−.275	.002
BNP (pg/mL)	−.450	<.001	−.191	.001	−.177	.002	−.177	.038

Note

Spearman correlation was performed to evaluate the relationships.

Abbreviations: BNP, brain natriuretic peptide; CK, creatine kinase; cTnI, cardiac troponin I; eGFR, estimated glomerular filtration rate; HBDH, hydroxybutyrate dehydrogenase; LDH, lactic dehydrogenase.

**TABLE 3:** Relationships between cardiac biomarkers and eGFR _Schwartz_ categories

Cardiac biomarkers	Total population		eGFR_Schwartz_ categories (mL/min/1.73 m^2^)			
			≥90		<90	
	*r*	*P* value	*r*	*P* value	*r*	*P* value
cTnI (ng/mL)	−.034	.617	.014	.868	−.097	.411
CK (IU/L)	−.263	<.001	−.249	.004	.070	.559
CK‐MB (ng/mL)	−.190	.006	−.031	.721	−.157	.189
LDH (IU/L)	−.079	.253	−.021	.804	−.094	.430
HBDH (IU/L)	−.080	.248	.061	.479	−.111	.354
BNP (pg/mL)	−.020	.808	−.048	.638	−.249	.065

Note

Spearman correlation was performed to evaluate the relationships.

Abbreviations: BNP, brain natriuretic peptide; CK, creatine kinase; cTnI, cardiac troponin I; eGFR, estimated glomerular filtration rate; HBDH, hydroxybutyrate dehydrogenase; LDH, lactic dehydrogenase.

A multivariate logistic regression analysis was performed with eGFR ≥ 90 mL/min/1.73 m^2^ as the reference. After adjustment for potential confounders, as compared with eGFR_CKD‐EPI_ ≥ 90 mL/min/1.73 m^2^, eGFR_CKD‐EPI_ 60 to <90 mL/min/1.73 m^2^ showed no significant higher CVD biochemistry biomarkers, but eGFR _CKD‐EPI_ < 60 mL/min/1.73 m^2^ remained associated with a 2.83 (1.08‐7.41) [ratio (95% CI)] times higher cTnI and a 6.50 (2.32‐18.22) [ratio (95% CI)] times higher HBDH, but not CK, CK‐MB, LDH, and BNP (Model 4, Table 4). In child, as compared with eGFR_Schwartz_ ≥ 90 mL/min/1.73 m^2^, eGFR_Schwartz_ < 90 mL/min/1.73 m^2^ revealed no significant higher CVD biochemistry biomarkers (Model 4, Table S2).

**TABLE 4:** Associations of eGFR_CKE‐EPI_ categories with biomarkers of cardiac injury

Biomarker	Model	eGFR_CKE‐EPI_ categories (mL/min/1.73 m^2^)^a^					
		≥90		60 to <90		<60	
		OR	95% CI	OR	95% CI	OR	95% CI
cTnI	1	Reference	NA	2.38	1.16‐4.88	11.22	5.58‐22.54
	2	Reference	NA	1.95	0.90‐4.22	9.22	4.34‐19.55
	3	Reference	NA	1.32	0.58‐2.97	2.81	1.09‐7.23
	4	Reference	NA	1.28	0.56‐2.93	2.83	1.08‐7.41
CK	1	Reference	NA	0.77	0.42‐1.41	1.07	0.50‐2.20
	2	Reference	NA	0.71	0.36‐1.42	0.96	0.43‐2.13
	3	Reference	NA	0.62	0.30‐1.25	0.41	0.14‐1.17
	4	Reference	NA	0.59	0.29‐1.21	0.40	0.14‐1.16
CK‐MB	1	Reference	NA	1.14	0.35‐3.78	2.75	0.82‐9.17
	2	Reference	NA	0.77	0.20‐3.01	1.83	0.46‐7.22
	3	Reference	NA	0.72	0.17‐2.99	0.97	0.16‐5.71
	4	Reference	NA	0.73	0.17‐3.14	1.02	0.16‐6.67
LDH	1	Reference	NA	0.98	0.60‐1.60	3.05	1.83‐5.09
	2	Reference	NA	1.05	0.61‐1.82	3.36	1.91‐5.90
	3	Reference	NA	0.91	0.51‐1.62	2.11	0.98‐4.52
	4	Reference	NA	0.92	0.51‐1.66	2.10	0.96‐4.58
HBDH	1	Reference	NA	1.84	0.87‐3.88	7.49	3.61‐15.54
	2	Reference	NA	2.42	1.08‐5.44	10.11	4.56‐22.38
	3	Reference	NA	2.01	0.86‐4.69	6.50	2.37‐17.86
	4	Reference	NA	2.09	0.89‐4.93	6.50	2.32‐18.22
BNP	1	Reference	NA	2.78	1.87‐4.11	7.84	4.93‐12.45
	2	Reference	NA	1.50	0.96‐2.35	4.76	2.87‐7.92
	3	Reference	NA	1.18	0.74‐1.91	1.83	0.92‐3.63
	4	Reference	NA	1.13	0.69‐1.85	1.91	0.95‐3.85

Abbreviations: BMI, body mass index; BNP, brain natriuretic peptide; CHD, coronary heart disease; CI, confidence interval; CK, creatine kinase; cTnI, cardiac troponin I;ECG, electrocardiogram; eGFR, estimated glomerular filtration rate; HBDH, hydroxybutyrate dehydrogenase; HDL‐C, high‐density lipoprotein‐cholesterol; LDL‐C, low‐density lipoprotein‐cholesterol; NA, not applicable; OR, odds ratio; TG, triglyceride.

^a^ Associations of eGFR_CKD‐EPI_ with cTnI, CK, CK‐MB, LDH, HBDH, and BNP were evaluated with logistic regression analysis. Model 1: unadjusted model; Model 2: age, gender, BMI, smoking and alcohol behavior; Model 3: model 2 + urea, TG, LDL‐C/HDL‐C, ST‐T wave abnormalities of ECG, previous CHD, previous CHD surgeries, hypertension and diabetes. Model 4: model 3 + antihypertensive medications, lipid‐modifying medications, antiplatelet drugs, and other heart diseases.

### 3.3 Additional analyses with eGFR _MDRD_

The median eGFR_MDRD_ was 89.25 mL/min/1.73 m^2^. Most participants had an eGFR_MDRD_ ≥ 90 mL/min/1.73 m^2^ (48.4%)or 60 to <90 mL/min/1.73 m^2^ (35.3%), while 16.3% had eGFR_MDRD_ < 60 mL/min/1.73 m^2^. Participants with lower eGFR_MDRD_ had significantly higher cTnI, CK, CK‐MB, LDH, HBDH, and BNP (Figure S6). Spearman correlation analysis showed negative correlations between eGFR_MDRD_ and cTnI (*r* = −.338; *P* < .001), CK (*r* = −.129; *P* < .001), CK‐MB (*r* = −.237; *P* < .001), LDH (*r* = −.153; *P* < .001), HBDH (*r* = −0.160; *P* < .001), and BNP (*r* = −.334; *P* < .001) (Table S3 and Figure S7). After adjustment for potential confounders, as compared with eGFR_MDRD_ ≥ 90 mL/min/1.73 m^2^, eGFR_MDRD_ 60 to <90 mL/min/1.73 m^2^ showed no significant higher CVD biochemistry biomarkers, but eGFR_MDRD_ < 60 mL/min/1.73 m^2^ remained associated with a 6.18 (2.49‐15.34) [ratio (95% CI)] HBDH (Model 4, Table S4). When eGFR _CKD‐EPI_ was replaced by eGFR_MDRD_, the associations of eGFR with cardiac biomarkers became weaker.

## 4 DISCUSSION

In a cross‐sectional study, we demonstrated that there were negative correlations between eGFR_CKD‐EPI_ and cTnI, CK‐MB, LDH, HBDH, and BNP. After adjustment for potential confounders, as compared with eGFR_CKD‐EPI_ ≥ 90 mL/min/1.73 m^2^, eGFR_CKD‐EPI_ < 60 mL/min/1.73 m^2^ remained associated with a 2.83 (1.08‐7.41) [ratio (95% CI)] times higher cTnI and a 6.50 (2.32‐18.22) [ratio (95% CI)] times higher HBDH. For child, there were negative correlations between eGFR_Schwartz_ and CK, and eGFR_Schwartz_ and CK‐MB. However, after adjustment for potential confounders, as compared with eGFR_Schwartz_ ≥ 90 mL/min/1.73 m^2^, eGFR_Schwartz_ < 90 mL/min/1.73 m^2^ revealed no significant higher CVD biomarkers.

cTn, which included cTnI and cTnT, are released following myocardial injury. Both cTn are used interchangeably in clinical practice, whereas cTnT has been suggested to be more strongly dependent on renal elimination than cTnI.^17^ Stronger associations of eGFR^18^, ^19^, ^20^ and measured GFR^21^ with cTnT than cTnI at levels < 60 mL/min/1.73 m^2^ were observed even when both cTn were measured with high sensitivity assays.^17^, ^18^, ^20^ However, Tuncay et al^13^ found that there was no significant relationship between eGFR and cTnI. In this study, we found that after adjustment for potential confounders, as compared with eGFR_CKD‐EPI_ ≥ 90 mL/min/1.73 m^2^, eGFR_CKD‐EPI_ < 60 mL/min/1.73 m^2^ remained associated with a 2.83 (1.08‐7.41) [ratio (95% CI)] times higher cTnI in adult participates. For child participates, eGFR_Schwartz_ < 90 mL/min/1.73 m^2^ showed no significant negative correlations as compared with eGFR_Schwartz_ ≥ 90 mL/min/1.73 m^2^.

The population with reduced eGFR were at the highest risk for CVD.^2^ There was a gradual and independent association between low eGFR and artery calcification, which is a well‐known predictor of CVD.^22^ The reduced eGFR may cause myocardial injury via chronic low‐grade inflammation and endothelial dysfunction,^23^ and may also reduce the renal elimination of cardiac biomarkers.^18^, ^24^ We could not determine the relative contributions of lower renal elimination and myocardial injury to the associations of eGFR with the cardiac biomarkers in our study. The negative correlations were also observed after adjustment for ST‐T wave abnormalities of ECG, which may indicate the lower renal elimination. However, the positive cardiac biomarkers may indicate minimal myocardial injury that is subclinical and not visible on an ECG. More and more studies^12^, ^23^ hold the opinion that myocardial injury involved, not only lower renal elimination. Thus, the results of our study suggested that minimal myocardial injury may contribute to the CVD mortality in the lower eGFR.

In this study, both eGFR_CKD‐EPI_ and eGFR_MDRD_ were associated with the cardiac biomarkers. Nevertheless, associations of eGFR_CKD‐EPI_ with cardiac biomarkers were stronger with eGFR_MDRD_. After adjustment for potential confounders, as compared with eGFR_CKD‐EPI_ ≥ 90 mL/min/1.73 m^2^, eGFR_CKD‐EPI_ < 60 mL/min/1.73 m^2^ remained associated with a 2.83 (1.08‐7.41) [ratio (95% CI)] times higher cTnI and a 6.50 (2.32‐18.22) [ratio (95% CI)] times higher HBDH. For eGFR_MDRD_, as compared with eGFR_MDRD_ ≥ 90 mL/min/1.73 m^2^, eGFR_MDRD_ < 60 mL/min/1.73 m^2^ only remained associated with a 6.18 (2.49‐15.34) [ratio (95% CI)] HBDH, but no cTnI. Previous study has clarified associations of eGFR with the cardiac biomarkers were stronger with GFR estimates that included cystatin C.^12^ Moreover, The CKD‐EPI equation is more accurate than the MDRD equation^14^ and combined creatinine‐cystatin C equation had greater precision and accuracy than the individual creatinine and cystatin C equation^25^ This phenomenon may indicate that associations of eGFR with cardiac biomarkers were stronger when calculated with more accurate equation.

This study has several limitations. On the one hand, owing to the cross‐sectional design, it is difficult for us to make strong causal inferences. On the other hand, this study was intrinsically limited by its retrospective nature. So, the associations between eGFR and cardiac biomarkers need additional exploration in future studies.

## CONFLICT OF INTERESTS

The author(s) declared no potential conflicts of interest with respect to the research, authorship, and/or publication of this article.

## ETHICAL APPROVAL

The research was in compliance with the Declaration of Helsinki and approved by the ethics committee of Peking University First Hospital (reference number: 1381).

## Supporting information

Fig S1

Fig S2

Fig S3

Fig S4

Fig S5

Fig S6

Fig S7

Table S1

Table S2

Table S3

Table S4
