# Supplementary material for: Associations between estimated glomerular filtration rate and cardiac biomarkers
Source: J Clin Lab Anal. 2020 Apr 16;34(8):e23336. doi: 10.1002/jcla.23336 (PMC7439334; doi:10.1002/jcla.23336)
Supplement: Supplementary file 8 — Table S1 [file JCLA-34-e23336-s008.docx]

Supplemental Table 1. Clinical characteristics of the children participants stratified according to eGFR _Schwartz_ categories. ^a^

|  | | | | Study population | eGFR _Schwartz_ categories (mL/min/1.73 m^2^) | | P values ^b^ |
| --- | --- | --- | --- | --- | --- | --- | --- |
|  |  |  |  |  | ≥ 90 | < 90 |  |
| Number | | | | 215 | 141 (65.6%) | 74 (34.4%) |  |
| Demographics | | | |  |  |  |  |
|  | Age (years) | | | 7.9 ± 5.0 | 8.2 ± 4.4 | 7.3 ± 5.8 | = 0.065 |
|  | Gender | | |  |  |  | = 0.875 |
|  |  | | Boys | 112 (52.1%) | 74 (52.5%) | 38 (51.4%) |  |
|  |  | | Girls | 103 (47.9%) | 67 (47.5%) | 36 (48.6%) |  |
|  | BMI (kg/m^2^) | | | 18.48 ± 4.61 | 18.57 ± 4.82 | 18.30 ± 4.22 | = 0.689 |
| Lifestyle variables | | | |  |  |  |  |
|  | Smoking behavior | | |  |  |  | = 0.166 |
|  |  | Never | | 214 (99.5) | 141 (100%) | 73 (98.6%) |  |
|  |  | Current | | 1 (0.5%) | 0 (0%) | 1 (1.4%) |  |
|  | Alcohol behavior | | |  |  |  | - |
|  |  | Never | | 215 (100%) | 141 (100%) | 90 (100%) |  |
|  |  | Current | | 0 (0%) | 0 (0%) | 0 (0%) |  |
| Lipid | | | |  |  |  |  |
|  | TG (mmol/L) | | | 1.04 (0.74-1.59) | 1.00 (0.70-1.49) | 1.10 (0.79-1.72) | = 0.166 |
|  | LDL-C/HDL-C | | | 2.06 (1.60-2.96) | 1.97 (1.51-2.62) | 2.17 (1.78-3.27) | = 0.028 |
| Medical history | | | |  |  |  |  |
|  | Previous CHD | | | 0 (0%) | 0 (0%) | 0 (0%) | - |
|  | Previous CHD surgeries ^c^ | | | 0 (0%) | 0 (0%) | 0 (0%) | - |
|  | Other heart diseases ^d^ | | | 15 (7.0%) | 9 (6.4%) | 6 (8.1%) | = 0.637 |
|  | Hypertension | | | 14 (6.5%) | 6 (4.3%) | 8 (10.8%) | = 0.119 |
|  | Diabetes | | | 2 (0.9%) | 1 (0.7%) | 1 (1.4%) | = 1.000 |
| Medications | | | |  |  |  |  |
|  | Antihypertensive medications ^e^ | | | 20 (9.3) | 12 (8.5%) | 8 (10.8%) | = 0.581 |
|  | Lipid-modifying medications ^f^ | | | 1 (0.5%) | 1 (0.7%) | 0 (0%) | = 1.000 |
|  | Antiplatelet drugs ^g^ | | | 4 (1.9%) | 3 (2.1%) | 1 (1.4%) | = 1.000 |
| ST-T wave abnormalities of ECG | | | | 24 (11.2%) | 18 (12.8%) | 6 (8.1%) | = 0.303 |
| Kidney biomarkers | | | |  |  |  |  |
|  | Creatine (μmol/L) | | | 53.32 ± 42.55 | 43.95 ± 11.22 | 71.29 ± 67.62 | < 0.001 |
|  | eGFR _Schwartz_ (mL/min/1.73 m^2^) | | | 99.93 ± 32.70 | 113.14 ± 31.00 | 74.77 ± 17.68 | < 0.001 |
|  | Urea (mmol/L) | | | 4.00 (3.16-5.04) | 3.92 (3.14-4.74) | 4.20 (3.17-6.37) | = 0.022 |
| Cardiac biomarkers | | | |  |  |  |  |
|  | cTnI (ng/mL) | | | 0.003 (0.001-0.006) | 0.003 (0.001-0.006) | 0.002 (0.001-0.005) | = 0.626 |
|  | CK (IU/L) ^h^ | | | 67 (47-104) | 63 (40-92) | 83 (55-125) | = 0.003 |
|  | CK-MB (ng/mL) ^i^ | | | 1.2 (0.7-2.1) | 1.1 (0.7-1.7) | 1.4 (0.8-2.4) | = 0.004 |
|  | LDH (IU/L) ^j^ | | | 252 (205-315) | 248 (202-314) | 255 (216-350) | = 0.290 |
|  | HBDH (IU/L) ^k^ | | | 197 (157-247) | 190 (153-24) | 215 (168-256) | = 0.079 |
|  | BNP (pg/mL) ^l^ | | | 16 (7-29) | 16 (7-29) | 12 (6-31) | = 0.606 |

^a^ Data are represented as means ± standard deviations for Gaussian distribution, medians (interquartile ranges) for non-Gaussian distribution and n (%) for categorical data.

^b^ *P* values for the comparison of participants between the eGFR categories were calculated with the *t* test for Gaussian distributed data, Mann-Whitney *U* test for non-Gaussian distribution and Chi-square (χ^2^) test for categorical data.

^c^ CHD surgeries include percutaneous coronary intervention and coronary artery bypass grafting.

^d^ Other heart diseases include cardiac arrhythmia, congenital cardiovascular diseases, cardiomyopathy, rheumatic heart disease, valvular heart disease, infective endocarditis and myocarditis.

^e^ Antihypertensive medications include angiotensin-converting enzyme inhibitors, angiotensin receptor blockers, calcium ion antagonists and β adrenoceptor blockers.

^f^ Lipid-modifying medications include statins, probucol and acipimox.

^g^ Antiplatelet drugs include aspirin, clopidogrel and ticagrelor.

^h^ Data available for 207 participants, including 135, 72 participants in three eGFR categories (≥ 90, < 90 mL/min/1.73 m^2^).

^i^ Data available for 212 participants, including 140, 72 participants in three eGFR categories (≥ 90, < 90 mL/min/1.73 m^2^).

^j^ Data available for 209 participants, including 137, 72 participants in three eGFR categories (≥ 90, < 90 mL/min/1.73 m^2^).

^k^ Data available for 208 participants, including 136, 72 participants in three eGFR categories (≥ 90, < 90 mL/min/1.73 m^2^).

^l^ Data available for 156 participants, including 100, 56 participants in three eGFR categories (≥ 90, < 90 mL/min/1.73 m^2^).

Abbreviation: BMI: body mass index; BNP: brain natriuretic peptide; CK: creatine kinase; cTnI: cardiac troponin I; CHD: coronary heart disease; ECG: electrocardiogram; eGFR: estimated glomerular filtration rate; HBDH: hydroxybutyrate dehydrogenase; HDL-C: high density lipoprotein cholesterol; LDL-C: low density lipoprotein cholesterol; LDH: lactic dehydrogenase; TG: triglyceride.
