# Supplementary material for: Associations between estimated glomerular filtration rate and cardiac biomarkers
Source: J Clin Lab Anal. 2020 Apr 16;34(8):e23336. doi: 10.1002/jcla.23336 (PMC7439334; doi:10.1002/jcla.23336)
Supplement: Supplementary file 9 — Table S2 [file JCLA-34-e23336-s009.docx]

Supplemental Table 2. Associations of eGFR _Schwartz_ with biomarkers of cardiac injury.

| Biomarker | Model | eGFR _Schwartz_ categories (mL/min/1.73 m^2^) ^a^ | | | |
| --- | --- | --- | --- | --- | --- |
|  |  | ≥ 90 | | < 90 | |
|  |  | OR | 95% CI | OR | 95% CI |
| cTnI | 1 | Reference | NA ^b^ | 3.578 | 1.012-12.650 |
|  | 2 | Reference | NA | 3.713 | 1.028-13.410 |
|  | 3 | Reference | NA | 1.942 | 0.462-8.172 |
|  | 4 | Reference | NA | 2.196 | 0.502-9.610 |
| CK | 1 | Reference | NA | 2.560 | 0.963-6.809 |
|  | 2 | Reference | NA | 2.561 | 0.933-7.027 |
|  | 3 | Reference | NA | 2.438 | 0.797-7.454 |
|  | 4 | Reference | NA | 2.376 | 0.785-7.189 |
| CK-MB | 1 | Reference | NA | 2.537 | 0.660-9.758 |
|  | 2 | Reference | NA | 1.969 | 0.472-8.211 |
|  | 3 | Reference | NA | 0.979 | 0.197-4.877 |
|  | 4 | Reference | NA | 1.024 | 0.200-5.236 |
| LDH | 1 | Reference | NA | 1.554 | 0.861-2.805 |
|  | 2 | Reference | NA | 1.428 | 0.724-2.815 |
|  | 3 | Reference | NA | 1.182 | 0.564-2.479 |
|  | 4 | Reference | NA | 1.065 | 0.499-2.274 |
| HBDH | 1 | Reference | NA | 1.545 | 0.855-2.792 |
|  | 2 | Reference | NA | 1.261 | 0.642-2.474 |
|  | 3 | Reference | NA | 1.023 | 0.485-2.161 |
|  | 4 | Reference | NA | 1.029 | 0.479-2.210 |
| BNP | 1 | Reference | NA | 5.389 | 1.368-21.234 |
|  | 2 | Reference | NA | 5.067 | 1.269-20.230 |
|  | 3 | Reference | NA | 1.347 | 0.221-8.209 |
|  | 4 | Reference | NA | 1.372 | 0.231-8.153 |

^a^ Associations of eGFR _Schwartz_ with cTnI, CK, CK-MB, LDH, HBDH and BNP were evaluated with logistic regression analysis. Model 1: unadjusted model; Model 2: age, gender, BMI, smoking behavior and alcohol behavior; Model 3: model 2 + urea, TG, LDL-C/HDL-C, ST-T wave abnormalities of ECG, previous CHD, previous CHD surgeries, hypertension and diabetes. Model 4: model 3 + antihypertensive medications, lipid-modifying medications, antiplatelet drugs and other heart diseases.

^b^ NA: not applicable.

Abbreviation: BMI: body mass index; BNP: brain natriuretic peptide; CHD: coronary heart disease; CK: creatine kinase; CI: confidence interval; cTnI: cardiac troponin I; ECG: electrocardiogram; eGFR: estimated glomerular filtration rate; HDL-C: high density lipoprotein cholesterol; HBDH: hydroxybutyrate dehydrogenase; LDL-C: low density lipoprotein cholesterol; OR: odds ratio; TG: triglyceride.
