# Supplementary material for: Associations between estimated glomerular filtration rate and cardiac biomarkers
Source: J Clin Lab Anal. 2020 Apr 16;34(8):e23336. doi: 10.1002/jcla.23336 (PMC7439334; doi:10.1002/jcla.23336)
Supplement: Supplementary file 10 — Table S3 [file JCLA-34-e23336-s010.docx]

Supplemental Table 3. Relationships between cardiac biomarkers and eGFR _MDRD_ categories. ^a^

| Cardiac biomarkers | Total population | | eGFR _MDRD_ categories (mL/min/1.73 m^2^) | | | | | |
| --- | --- | --- | --- | --- | --- | --- | --- | --- |
|  |  |  | ≥ 90 | | 60 to < 90 | | < 60 | |
|  | r | P value | r | P value | r | P value | r | P value |
| cTnI (ng/mL) | -0.338 | < 0.001 | 0.024 | = 0.639 | -0.201 | = 0.001 | -0.184 | = 0.035 |
| CK (IU/L) | -0.129 | < 0.001 | -0.158 | = 0.002 | 0.056 | = 0.363 | 0.061 | = 0.508 |
| CK-MB (ng/mL) | -0.237 | < 0.001 | -0.130 | = 0.011 | -0.150 | = 0.014 | -0.043 | = 0.643 |
| LDH (IU/L) | -0.153 | < 0.001 | -0.071 | = 0.173 | 0.022 | = 0.723 | -0.211 | = 0.020 |
| HBDH (IU/L) | -0.160 | < 0.001 | -0.045 | = 0.394 | 0.010 | = 0.877 | -0.256 | = 0.006 |
| BNP (pg/mL) | -0.334 | < 0.001 | 0.027 | = 0.622 | -0.216 | = 0.001 | -0.187 | = 0.039 |

^a^ Spearman correlation was performed to evaluate the relationships.

Abbreviation: BNP: brain natriuretic peptide; CK: creatine kinase; cTnI: cardiac troponin I; eGFR: estimated glomerular filtration rate; HBDH: hydroxybutyrate dehydrogenase; LDH: lactic dehydrogenase.
