# Supplementary material for: Associations between estimated glomerular filtration rate and cardiac biomarkers
Source: J Clin Lab Anal. 2020 Apr 16;34(8):e23336. doi: 10.1002/jcla.23336 (PMC7439334; doi:10.1002/jcla.23336)
Supplement: Supplementary file 11 — Table S4 [file JCLA-34-e23336-s011.docx]

Supplemental Table 4. Associations of eGFR _MDRD_ categories with biomarkers of cardiac injury.

| Biomarker | Model | eGFR _MDRD_ categories (mL/min/1.73 m^2^) ^a^ | | | | | |
| --- | --- | --- | --- | --- | --- | --- | --- |
|  |  | ≥ 90 | | 60 to < 90 | | < 60 | |
|  |  | OR | 95% CI | OR | 95% CI | OR | 95% CI |
| cTnI | 1 | Reference | NA ^b^ | 2.20 | 1.17-4.14 | 8.94 | 4.83-16.54 |
|  | 2 | Reference | NA | 1.80 | 0.93-3.47 | 7.85 | 4.17-14.79 |
|  | 3 | Reference | NA | 1.32 | 0.66-2.64 | 2.34 | 1.00-5.47 |
|  | 4 | Reference | NA | 1.26 | 0.62-2.57 | 2.30 | 0.97-5.48 |
| CK | 1 | Reference | NA | 0.90 | 0.49-1.63 | 1.07 | 0.51-2.27 |
|  | 2 | Reference | NA | 0.79 | 0.42-1.50 | 0.96 | 0.44-2.08 |
|  | 3 | Reference | NA | 0.72 | 0.37-1.38 | 0.37 | 0.12-1.15 |
|  | 4 | Reference | NA | 0.70 | 0.36-1.36 | 0.35 | 0.11-1.10 |
| CK-MB | 1 | Reference | NA | 1.42 | 0.45-4.46 | 2.66 | 0.80-8.87 |
|  | 2 | Reference | NA | 0.96 | 0.29-3.18 | 2.00 | 0.57-6.96 |
|  | 3 | Reference | NA | 1.05 | 0.31-3.57 | 1.06 | 0.19-6.03 |
|  | 4 | Reference | NA | 1.07 | 0.31-3.76 | 0.97 | 0.15-6.33 |
| LDH | 1 | Reference | NA | 0.91 | 0.55-1.50 | 3.31 | 2.01-5.46 |
|  | 2 | Reference | NA | 0.91 | 0.54-1.53 | 3.40 | 2.03-5.68 |
|  | 3 | Reference | NA | 0.85 | 0.49-1.48 | 2.29 | 1.11-4.69 |
|  | 4 | Reference | NA | 0.84 | 0.48-1.46 | 2.31 | 1.11-4.82 |
| HBDH | 1 | Reference | NA | 1.63 | 0.80-3.32 | 7.96 | 4.09-15.51 |
|  | 2 | Reference | NA | 1.79 | 0.85-3.77 | 8.73 | 4.38-17.41 |
|  | 3 | Reference | NA | 1.60 | 0.74-3.47 | 6.12 | 2.51-14.93 |
|  | 4 | Reference | NA | 1.56 | 0.71-3.43 | 6.18 | 2.49-15.34 |
| BNP | 1 | Reference | NA | 2.16 | 1.51-3.11 | 5.29 | 3.40-8.24 |
|  | 2 | Reference | NA | 1.47 | 0.99-2.18 | 4.62 | 2.86-7.47 |
|  | 3 | Reference | NA | 1.21 | 0.79-1.83 | 1.69 | 0.88-3.28 |
|  | 4 | Reference | NA | 1.12 | 0.73-1.73 | 1.82 | 0.93-3.57 |

^a^ Associations of eGFR _MDRD_ with cTnI, CK, CK-MB, LDH, HBDH and BNP were evaluated with logistic regression analysis. Model 1: unadjusted model; Model 2: age, gender, BMI, smoking behavior and alcohol consumption; Model 3: model 2 + urea, TG, LDL-C/HDL-C, ST-T wave abnormalities of ECG, previous CHD, previous CHD surgeries, hypertension and diabetes. Model 4: model 3 + antihypertensive medications, lipid-modifying medications, antiplatelet drugs and other heart diseases.

^b^ NA: not applicable.

Abbreviation: BMI: body mass index; BNP: brain natriuretic peptide; CHD: coronary heart disease; CK: creatine kinase; CI: confidence interval; cTnI: cardiac troponin I; ECG: electrocardiogram; eGFR: estimated glomerular filtration rate; HDL-C: high density lipoprotein cholesterol; HBDH: hydroxybutyrate dehydrogenase; LDL-C: low density lipoprotein cholesterol; OR: odds ratio; TG: triglyceride.
